# Inflammation-mediated muscle metabolic dysregulation local and remote to the site of major abdominal surgery

**DOI:** 10.1016/j.clnu.2017.10.020

**Published:** 2018-12

**Authors:** Krishna K. Varadhan, Dumitru Constantin-Teodosiu, Despina Constantin, Paul L. Greenhaff, Dileep N. Lobo

**Affiliations:** a Gastrointestinal Surgery, Nottingham Digestive Diseases Centre, National Institute of Health Research (NIHR) Nottingham Biomedical Research Centre, Nottingham University Hospitals and University of Nottingham, Queen's Medical Centre, Nottingham NG7 2UH, UK; b MRC/ARUK Centre for Musculoskeletal Ageing Research, National Institute of Health Research (NIHR) Nottingham Biomedical Research Centre, School of Life Sciences, University of Nottingham, Queen's Medical Centre, Nottingham NG7 2UH, UK

**Keywords:** Abdominal surgery, Muscle inflammatory responses, Postoperative hyperglycaemia, Cytokines, Metabolic response, Gene expression, CSTL1, cathepsin, FFA, free fatty acid, FOXO, forkhead box O, IL, interleukin, IR, insulin resistance, IRS1, insulin receptor substrate 1, LPS, lipopolysaccharide, MAFbx, muscle atrophy F-box, MuRF1, muscle-RING finger 1, PCA, perchloric acid, PDC, pyruvate dehydrogenase complex, PDK, pyruvate dehydrogenase kinase, PI3K/Akt1, phosphotidyl inositol 3-kinase/protein kinase B, PPAR, perioxisome proliferator-activated receptor, RA, rectus abdominus, TNF-α, tumour necrosis factor α, VL, vastus lateralis

## Abstract

**Background & aims:**

Postoperative hyperglycaemia is common in patients having major surgery and is associated with adverse outcomes. This study aimed to determine whether bacteraemia contributed to postoperative systemic inflammation, and whether increases in the expression of muscle mRNAs and proteins reflecting increased muscle inflammation, atrophy and impaired carbohydrate oxidation were evident at the time of surgery, and both local and distant to the site of trauma, and could be associated with impaired glucoregulation.

**Methods:**

Fifteen adult patients without diabetes undergoing major abdominal surgery participated in this observational study set in a university teaching hospital. Arterialised-venous blood samples and muscle biopsies were obtained before and after major elective abdominal surgery, from sites local (rectus abdominis – RA) and remote to the site of surgery (vastus lateralis – VL). The main outcome measures included blood glucose concentrations, gut permeability and changes in expression of muscle mRNAs and proteins linked to inflammation and glucose regulation.

**Results:**

Immediately postoperatively, RA demonstrated markedly increased mRNA expression levels of cathepsin-L (7.5-fold, *P* < 0.05), FOXO1 (10.5-fold, *P* < 0.05), MAFbx (11.5-fold, *P* < 0.01), PDK4 (7.8-fold, *P* < 0.05), TNF-α (16.5-fold, *P* < 0.05) and IL-6 (1058-fold, *P* < 0.001). A similar, albeit blunted, response was observed in VL. Surgery also increased expression of proteins linked to inflammation (IL-6; 6-fold, *P* < 0.01), protein degradation (MAFbx; 4.5-fold, *P* < 0.5), and blunted carbohydrate oxidation (PDK4; 4-fold, *P* < 0.05) in RA but not VL. Increased systemic inflammation (TNF-α, *P* < 0.05; IL-6, *P* < 0.001), and impaired postoperative glucose tolerance (*P* < 0.001), but not bacteraemia (although gut permeability was increased significantly, *P* < 0.05) or increased plasma cortisol, were noted 48 h postoperatively.

**Conclusions:**

A systemic postoperative proinflammatory response was accompanied by muscle inflammation and metabolic dysregulation both local and remote to the site of surgery, and was not accompanied by bacteraemia.

**Clinical trial registration:**

Registered at http://clinicaltrials.gov (NCT01134809).

## 1 Introduction

Skeletal muscle is the primary site for insulin-dependent glucose uptake [1], and along with the liver, plays an important role in the development of insulin resistance (IR) and hyperglycaemia during states of metabolic stress. One of the hallmarks of the response to trauma is the development of whole-body IR, which is proportional to the magnitude of injury [2]. IR is associated with raised blood glucose concentrations, and linked to this, increased risk of complications, particularly infectious ones, impaired wound healing, and delayed discharge from the hospital.

The metabolic response to major surgery, like that to trauma, starts as a local response, and evolves into a systemic one. The effectors of the systemic response are complex and involve interactions between cytokines, neurohormonal mediators and muscle. This metabolic response to major surgery may be exacerbated further by inflammation-related changes in gut permeability and resultant bacterial translocation [3]. Furthermore, it is unclear to what extent the effects of surgical stress at the site of the incision (e.g. abdominal muscles) are mirrored in tissues distant from the site of the injury (e.g. lower limb muscles). Significantly increased expression of interleukin-6 (IL-6), IL-6 receptor and tumour necrosis factor α (TNF-α) mRNA has been demonstrated in skeletal muscle local to the site of injury in non-septic post-surgery patients [4], but whether these gene transcript changes are accompanied by increases at the protein level and with metabolic dysregulation is unknown.

The reduction of muscle phosphotidyl inositol 3-kinase/protein kinase B (PI3K/Akt1) phosphorylation, ensuing the activation of downstream forkhead box O (FOXO) transcription factors, resulted in a simultaneous induction of muscle atrophy response and impairment of muscle carbohydrate oxidation within 24 h of lipopolysaccharide (LPS) infusion in a rodent model of clinical endotoxaemia [5]. Importantly, these events were accompanied by marked upregulation of FOXO downstream gene targets, including muscle atrophy F-box (MAFbx), muscle-RING finger 1 (MuRF1; a muscle specific E3-ligase that has been proposed to be a prerequisite for proteasome mediated proteolysis [6], [7]) and pyruvate dehydrogenase kinase-4 (PDK4) that phosphorylates and inactivates pyruvate dehydrogenase complex (PDC), the rate-limiting step in carbohydrate oxidation [8], [9]. Collectively, these data point to a role for Akt/FOXO signalling in the simultaneous induction of muscle atrophy and impairment of muscle carbohydrate oxidation during endotoxaemia. This suggestion was further substantiated by the observation that low-dose dexamethasone infusion, or peroxisome proliferator-activated receptor γ (PPARγ) agonism, during endotoxaemia both dampen muscle inflammation and cytokine-mediated upregulation of Akt/FOXO signalling, thereby reducing muscle atrophy and the impairment of carbohydrate oxidation in a rodent model of clinical endotoxaemia [10], [11].

In the context of this background, the aims of this observational study in patients undergoing major abdominal surgery were to determine whether:

(i) in addition to surgical trauma, bacteraemia secondary to altered gut permeability following major abdominal surgery contributed to post-operative systemic inflammation.
(ii) increases in the expression of muscle mRNAs and proteins representing muscle inflammation and activation of muscle protein degradation and inhibition of muscle carbohydrate oxidation, and previously observed in rodent model of clinical endotoxaemia [10], [12], were evident at the time of major abdominal surgery both local [rectus abdominis (RA) muscle] and distant [vastus lateralis (VL) muscle] to the site of surgical trauma, and were associated with impaired glucoregulation 48 h later.

## 2 Methods

### 2.1 Participants

Fifteen participants undergoing major elective open abdominal surgery were studied, following national research ethics service approval and informed written consent. The protocol was registered at http://clinicaltrials.gov (NCT01134809). Haemoglobin, blood urea and electrolytes, and liver function tests were assayed at screening. Exclusion criteria included emergency surgery, liver surgery, chronic illness (e.g. diabetes mellitus), disseminated malignant disease, and medications known to affect glucose metabolism, statins, and chronic use of antibiotics, anti-inflammatory drugs, or immunosuppressive agents.

### 2.2 Protocol

Arterialised-venous blood samples obtained in the fasted state at the preassessment visit (screening), immediately before surgery, at the end of surgery and 48 h postoperatively were analysed for plasma concentrations of insulin, cytokines (IL-6 and TNF-α), cortisol and free fatty acid (FFA).

At the beginning and end of surgery, a biopsy was obtained from RA muscle from the area of the laparotomy wound, and from the VL muscle using a percutaneous conchotome. The VL specimens obtained at the end of surgery were from the contralateral limb. All muscle samples were stored in liquid nitrogen until analysis.

Glucose tolerance was assessed during the screening visit and 48 h postoperatively. On each occasion, a venous blood sample was obtained before (fasting) and 2 h following ingestion of 75 g of glucose (post-prandial).

A ^51^Cr-EDTA gut permeability test was performed during the screening visit and 48 h postoperatively using an oral preparation containing 1.8 MBq of ^51^Cr labelled EDTA (GE Healthcare, Amersham, UK) in 100 ml of water. The patients were not allowed to eat or drink for the next 2 h. Twenty-four-hour urine samples were collected during the screening visit and 48 h postoperatively for the ^51^Cr-EDTA tests. Whole blood samples collected 48 h postoperatively were used to determined levels of bacterial DNA.

### 2.3 Analyses

#### 2.3.1 Blood glucose and plasma insulin and free fatty acid concentrations

Whole blood glucose concentrations were determined using a HemoCue analyser (HemoCue AB, Ängelholm, Sweden), plasma insulin concentrations using an enzyme-linked immunosorbent assay (ELISA; Mercodia, Uppsala, Sweden), and plasma FFA concentrations using a NEFA-HR (2) R2 WAKO kit (Wako Chemicals GmbH, Germany) according to the manufacturer's instructions.

#### 2.3.2 Plasma cytokine and cortisol concentrations and blood bacterial DNA expression

Plasma IL-6, TNF-α and cortisol concentrations were determined using ELISA quantikine kits (R&D Systems, Minneapolis, USA) according to the manufacturer's recommendations.

Total blood DNA was isolated using a Qiagen DNeasy (Valencia, CA, USA) kit according to the manufacturer's recommendations. DNA was then quantified and used to identify levels of bacterial 16S rDNA using Taqman qPCR with the following nucleotide sequences: Forward primer: 5′-AACTGGAGGAAGGTGGGGAT-3′; Reverse primer: 5′-AGGAGGTGATCCAACCGCA-3′ and TaqMan probe: 5′-(6-FAM)-TACAAGGCCCGGGAACGTATTCACCG-(TAMRA)-3′. The captured sequence covered a 380-bp amplicon, which resides within the highly conserved C8 and C9 regions of the bacterial *16S rDNA* target [3], [13]. The positive control comprised DNA isolated from the Gram positive wild-type strain SG38 of *Bacillus subtilis*, which is found in the gastrointestinal tract of humans.

#### 2.3.3 Gut permeability

Urine samples were analysed in triplicate for ^51^Cr-EDTA radioactivity using a γ-scintillation counter along with an appropriate standard. Results were expressed as the percentage urinary excretion of the orally administered dose of ^51^Cr-EDTA after correction for background radioactivity and radioactive decay.

#### 2.3.4 Glycogen and lactate content in rectus abdominis and vastus lateralis muscles

A portion of each frozen RA and VL muscle sample was freeze-dried, and following the removal of all visible blood and connective tissue, was powdered. Muscle metabolites were extracted from 4 to 6 mg of muscle powder in 0.5 mmol/L perchloric acid (PCA, containing 1 mmol/L EDTA) and the following centrifugation, extracts were neutralised with 2.2 mmol/L KHCO_3_. Neutralised PCA extracts were also used to determine muscle lactate content using a modified spectrophotometric version of the method of Harris et al. [14] to accommodate the use of a plate reader. Additionally, 1 to 2 mg of freeze-dried muscle was alkaline extracted and muscle glycogen content was determined spectrophotometrically [14].

#### 2.3.5 Targeted mRNA expression levels in rectus abdominis and vastus lateralis muscles

mRNA expression of a selection of genes intimately linked to inflammation (IL-6, TNF-α), muscle protein degradation [ubiquitin E3-ligases, MaFbx and MURF1, cathepsin (CSTL1)], muscle growth (myostatin, or growth differentiation factor 8 MSTN) and insulin signalling and carbohydrate oxidation (insulin receptor substrate 1, IRS1; serine–threonine protein kinase B, AKT1; Forkhead Box O1, FOXO1; and pyruvate dehydrogenase kinase isoform 4, PDK4) was determined in RA and VL muscles.

Total RNA was extracted from approximately 25 mg of wet RA and VL muscle using Tri Reagent (Ambion, Fisher Scientific, Loughborough, UK), and subsequently quantified spectrophotometrically at 260 nm with RNA purity being determined as the ratio of 260/280 nm readings. Thereafter, first strand cDNA synthesis was carried out according to previous methodology [15]. mRNA expression levels of the targeted transcripts were determined using TaqMan PCR (ABI prism 7900HT sequence detector, Applied Biosystems, Warrington, UK), with 2 μl of cDNA, 18 μM of each primer, and 5 μM probe and Universal TaqMan 2× PCR Mastermix (Applied Biosystems, Warrington, UK). Each sample was run in duplicate. Primers and MGB TaqMan probes (Applied Biosystems, Warrington, UK) were designed such that probes spanned over exon–exon boundaries to avoid genomic amplification. Hydroxymethylbilane synthase (HMBS) was used as an internal control, and all genes of interest were labelled with the fluorescent reporter 5-FAM (5-Carboxyfluorescein). The thermal cycling conditions used were: 2 min at 50 °C, 10 min at 95 °C, followed by 40 cycles at 95 °C for 15 s and 60 °C for 1 min.

#### 2.3.6 Targeted protein expression levels in rectus abdominis and vastus lateralis muscles

Proteins expression levels of IL-6, MaFbx, p70S6 kinase, FOXO1 and PDK4 were determined in RA and VL muscle by Western blotting. A portion of frozen wet muscle was homogenised in Tris buffer (Tris 50 mM/EDTA 1 mM pH 7.5) that included protease and phosphatase inhibitors (Sigma–Aldrich, St Louis, Missouri, USA). After homogenisation, each muscle extract was centrifuged for 15 min at 10,000 *g*. The supernatant was collected and stored at −80 °C for further protein immunoblotting [15]. Protein concentration was measured using the Bradford assay. Protein samples were run on a 4–12% Bis–Tris acrylamide gel (Invitrogen, Carlsbad, CA, USA) for 2 h at constant 200 V and transferred to a polyvinylidene difluoride membrane (PVDF) overnight (at a constant 100 mA), in ice-cold buffers (4 °C). The protein transfer was checked with Ponceau S red staining, before blocking the membrane in BSA, TBS, and Tween for 1 h at room temperature. Membranes were probed with the primary antibody overnight at 4 °C. Antibodies used [phosphorylated (Ser^256^ PFOXO1) and total FOXO1, phosphorylated p70 ribosomal S6 kinase (Thr^389^ Pp70S6k) (Pp70S6k) and total p70S6k (p70S6k), IL-6 and α-actin] were obtained from Cell Signaling Technology (Danvers, MA, USA). MAFbx, produced in-house by Pfizer Inc. (USA), and PDK4 produced in-house by AstraZeneca (UK), were provided as gifts. Membranes were washed with TBS-T and incubated with the appropriate LICOR IR secondary antibody. The membranes were washed again in the same buffer and the bands were quantified using an IR-detection LICOR Odyssey system.

### 2.4 Statistical analysis

Data in text, tables and figures are expressed as mean ± SEM or median (interquartile range) where appropriate and in accordance with accepted convention (*n* = 15). Differences between pre- and postoperative blood and muscle metabolites were determined using the Student's t test for paired samples (for parametric data) and related samples Wilcoxon Signed Ranks Test (for non-parametric data). The difference in 2 h post-prandial blood glucose concentration between screening and 48 h postoperatively was determined using the Chi-square test. Differences were considered significant at *P* < 0.05.

## 3 Results

There were 15 participants (13 male) with a mean ± SEM age of 49.0 ± 4.5 yrs and body mass index of 26.0 ± 1.7 kg/m^2^. Seven were operated for cancer and 8 for benign diseases, such as chronic pancreatitis and large incisional herniae. Mean time elapsed between the start and the end of surgery was 223 ± 22 min (range 110–390 min).

### 3.1 Clinical and laboratory tests

Haemoglobin, blood urea and electrolytes, and liver function tests measured during the screening visit were within the normal laboratory ranges for all participants.

#### 3.1.1 Blood glucose and plasma insulin, cytokines, cortisol and free fatty acid concentrations

Table 1 shows that, according to the ranges suggested by the World Health Organisation [16], significantly more participants had higher fasting and post-prandial blood glucose concentrations postoperatively when compared with the screening test (*P* < 0.001).

**Table 1:** Fasting and 2 h post-prandial blood glucose concentration determined at the volunteer screening visit and 48 h postoperatively. Values represent the number of participants falling into each blood glucose concentration range according to the WHO definition [16].

Blood glucose (mmol/L)	Screening visit		48 h postoperatively	
	Fasting	2 h post-prandial	Fasting	2 h post-prandial
<6.1	13	9	12	1
≥6.1 & <7.0	0	0	3	0
≥7.0 & <7.8	1	1	0	1
≥7.8	1	5	0	13

Screening *vs.* 48 h postoperatively *P* < 0.001; Chi-square test.

Compared with the screening visit, fasting plasma insulin concentration was significantly lower at the start (*P* < 0.05) and the end (*P* < 0.05) of surgery, but were no different 48 h postoperatively (Table 2). Plasma IL-6 concentration was 15-fold greater at the end of surgery compared with screening (*P* < 0.001) and remained elevated 48 h postoperatively (*P* < 0.001, Table 2). Plasma TNF-α concentration was also elevated 48 h postoperatively compared with screening (*P* < 0.05; Table 2), but this difference was not of the very large magnitude seen for plasma IL-6 concentration. Plasma cortisol concentration was elevated 3.5-fold immediately after surgery compared with screening (*P* < 0.001), but was no different from screening 48 h postoperatively (Table 2). Plasma FFA concentration was greater than at screening at all time-points, with the difference being most apparent immediately after surgery (3-fold, *P* < 0.001; Table 2).

**Table 2:** Fasted plasma insulin, cytokines, cortisol and free fatty acids concentrations in patients undergoing major elective abdominal surgery at screening, at the start of surgery, immediately after surgery and 48 h postoperatively (*n* = 15).

	Screening	At start of surgery	Immediately after surgery	48 h postoperatively
Insulin (mIU/L)	11.9 (8.3–18.4)	10.5 (5.5–15.4)^∗^	7.0 (5.7–14.2)^∗^	10.2 (6.0–18.3)
IL-6 (pg/mL)	3.4 (2.2–4.6)	3.12 (2.6–4.0)	21.8 (14.0–90.5)^∗∗∗^	31.7 (14.8–70.5)^∗∗∗^
TNF-α (pg/mL)	13.0 (11.5–14.2)	13.5 (12.5–14.2)	13.5 (12.9–14.2)	14.6 (12.3–16.1)^∗^
Cortisol (ng/mL)	1.8 (1.2–3.3)	2.1 (1.4–3.5)	8.1 (3.9–11.1)^∗∗∗^	2.9 (1.0–4.0)
Free fatty acids (mmol/L)	0.2 (0.1–0.3)	0.5 (0.3–0.7)^∗^	0.6 (0.4–1.0)^∗∗∗^	0.4 (0.2–0.6)^∗^

Data are expressed as median (interquartile range).

*P < 0.05; **P < 0.01; ***P < 0.001 significantly different from screening.

#### 3.1.2 Gut permeability and blood bacterial DNA expression

**Fig. 1:**
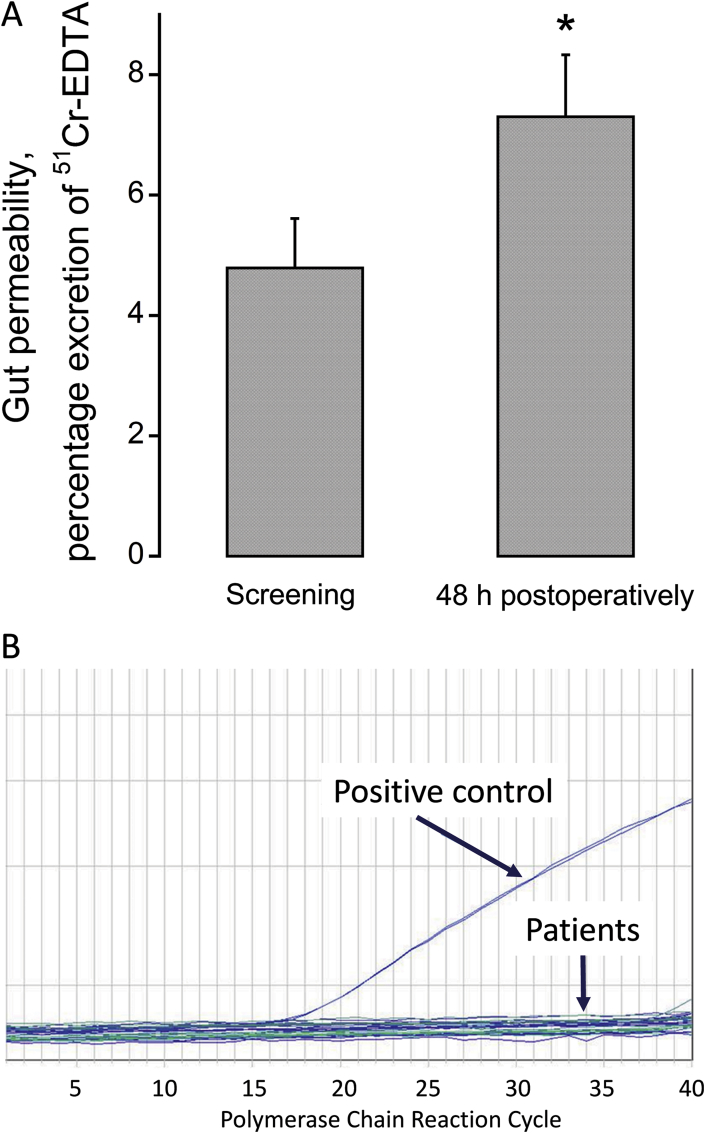
A. Gut permeability in participants undergoing major abdominal surgery preoperatively and 48 h postoperatively (*n* = 15), *P < 0.05. B. The presence of DNA representative of Gram positive bacteria in circulating blood in participants 48 h postoperatively (*n* = 15). The positive control comprised of DNA isolated from the Gram positive wild-type strain SG38 of *Bacillus subtilis*, which is found in the gastrointestinal tract of humans.

Gut permeability was increased 48 h postoperatively compared with the screening visit (Fig. 1A, *P* < 0.05). However, the presence of bacterial DNA in blood was undetectable 48 h postoperatively (Fig. 1B). Bacterial DNA was confirmed to be present in the positive control DNA (Fig. 1B), which was isolated from the Gram positive wild-type strain SG38 and is known to be found in the gastrointestinal tract of humans.

#### 3.1.3 Glycogen and lactate content in rectus abdominis and vastus lateralis muscles

Muscle glycogen and lactate content at the start of surgery and immediately after surgery in RA and VA are presented in Table 3. Following surgery, muscle glycogen content was less than that at the start of surgery in RA (*P* < 0.05), while muscle lactate was greater (*P* < 0.01). No differences were seen when comparing muscle glycogen and lactate contents of the VL at the start of surgery with those obtained at the end of surgery.

**Table 3:** Glycogen and lactate content in rectus abdominis and vastus lateralis muscles at the start of surgery and immediately after surgery in patients undergoing major elective abdominal surgery (*n* = 15).

Metabolite	Rectus abdominis muscle (local to the site of surgery)		Vastus lateralis muscle (distant from the site of surgery)	
	At start of surgery	Immediately after surgery	At start of surgery	Immediately after surgery
Glycogen (mmol/kg dry matter)	330 (300–361)	295 (275–320)*	341 (311–352)	337 (311–360)
Lactate (mmol/kg dry matter)	4.6 (3.4–5.6)	9.7 (8.9–11.2)**	5.5 (4.9–5.8)	5.98 (5.3–6.3)

Data are expressed as median (interquartile range).

*P < 0.05, **P < 0.01 significantly different from value at the start of surgery.

#### 3.1.4 mRNA expression levels in rectus abdominis and vastus lateralis muscles

**Fig. 2:**
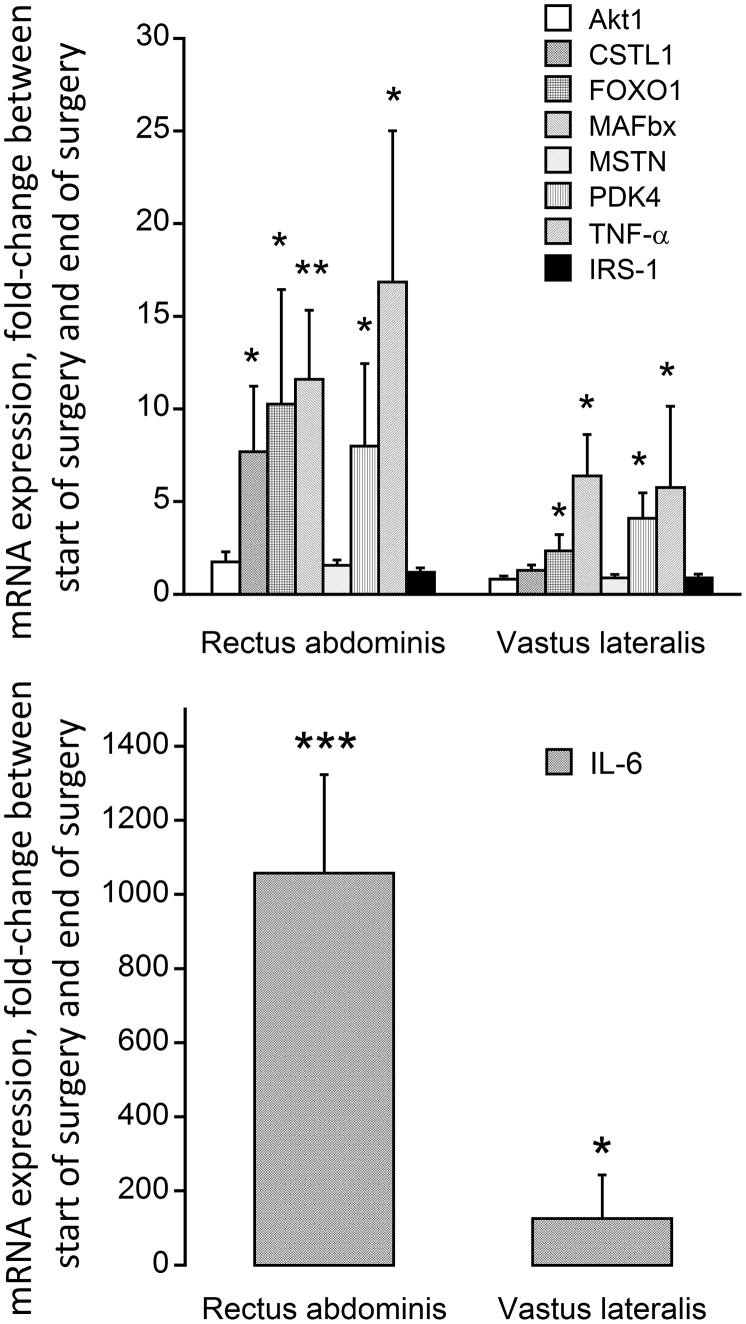
Fold change in (top) Akt1, Cathepsin-L (CSTL1), FOXO1, MAFbx, myostatin (MSTN), PDK4, TNF-α and IRS1 mRNA expression and (bottom) IL-6 mRNA expression in rectus abdominis (local to the site of surgery) and vastus lateralis (distant from the site of surgery site) muscle obtained at the start of surgery and immediately after surgery from participants undergoing major abdominal surgery (*n* = 15). Values are expressed as fold changes from the value at the start of surgery. *P < 0.05, **P < 0.01, ***P < 0.001, significantly different from the value at the start of surgery.

The mean change in mRNA abundance from the start of surgery to immediately after surgery was sizeable for cathepsin-L (CSTL 1; 7.5-fold, *P* < 0.05), FOXO1 (10.5-fold, *P* < 0.05), MAFbx (11.5-fold, *P* < 0.01), PDK4 (7.8-fold, *P* < 0.05) and TNF-α (16.5-fold, *P* < 0.05) in RA (Fig. 2A). No change in mRNA abundance was recorded for Akt 1, myostatin and IRS1 in RA. A similar pattern of change was observed in the VL, albeit of a lower magnitude, i.e. FOXO1 (2.5-fold, *P* < 0.05), MAFbx (6.4-fold, *P* < 0.05), PDK4 (4.1-fold, *P* < 0.05) and TNF-α (5.8-fold, *P* < 0.05; Fig. 2A), with no change in CSTL 1, myostatin, and IRS-1 abundance being observed. IL-6 demonstrated the most striking change in mRNA abundance from the start of surgery to immediately after surgery (1058- and 126-fold in the RA and VL, respectively; Fig. 2B).

#### 3.1.5 Targeted protein expression levels in rectus abdominis and vastus lateralis muscles

**Fig. 3:**
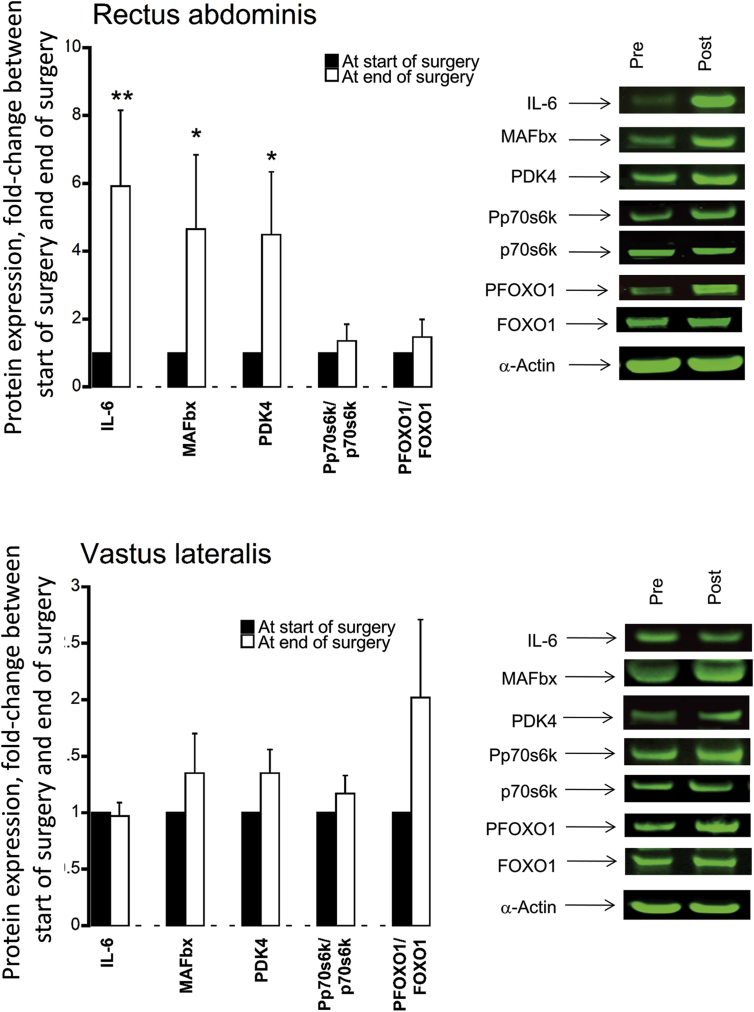
Relative protein expression levels of IL-6, MAFbx, PDK4, the ratio of Pp70S6K/p70S6K and the ratio of PFOXO1/FOXO1 in (top) rectus abdominis muscle (local to the site of surgery) and (bottom) vastus lateralis muscle (distant from the site of surgery) obtained at the start of surgery and immediately after surgery from participants undergoing major abdominal surgery (*n* = 15). Values are expressed as fold change from the value at the start of surgery. *P < 0.05, **P < 0.01 significantly different from the value at the start of surgery (set at 1).

At the end of surgery, protein expression levels of IL-6 (6-fold, P < 0.01), MAFbx (4.5-fold, *P* < 0.05) and PDK4 (4-fold, *P* < 0.05) in the RA were greater than at the start of surgery (Fig. 3A). No difference in protein abundance for the same proteins was recorded between the start of surgery to immediately after surgery in VL (Fig. 3B).

## 4 Discussion

The major findings of the present study were that surgical trauma induced a systemic inflammatory response immediately after surgery that was accompanied by an increase in gut permeability, but there was no evidence of bacteraemia or substantive changes in fasting blood glucose or plasma insulin and cortisol concentrations 48 h later. Post-prandial glucoregulation was, however, impaired 48 h postoperatively. Immediately after surgery, the abundance of mRNAs associated with tissue inflammation, protein breakdown and impaired carbohydrate oxidation was profoundly increased in muscle local to the site of surgical trauma (RA), and was mirrored by similar pattern of mRNA response in muscle distant to the site of injury (VL), albeit to a lesser extent. These muscle gene responses were parallelled by changes at the muscle protein level, and by lower muscle glycogen and elevated muscle lactate concentrations, at the site local to surgical trauma, but not in muscle away from the site of surgery. These findings collectively demonstrate that a systemic postoperative inflammatory response was accompanied by muscle inflammation and metabolic dysregulation both local and remote to the site of surgery, which is most marked at the site of surgery. These muscle level responses, and not bacteraemia, likely contribute to the impairment of muscle mass maintenance and systemic blood glucose regulation in the days following surgery.

Postoperative hyperglycaemia is an important component of post-operative complications and delayed recovery [17], [18] and arises as a result of decreased insulin-stimulated whole-body glucose disposal as well as increased endogenous glucose release [19]. Systemic inflammation has been reported to be an important driver of insulin resistance (including muscle insulin resistance, the largest contributor to whole-body glucose disposal) in ageing [20], [21], obesity [22], [23], postoperative stress [22], [24], [25], and also in muscle atrophy [20]. In keeping with this, a significant correlation between insulin resistance and plasma concentrations of IL-6 has been reported in patients undergoing open cholecystectomy and hernia repair [24], with only minor increments in the levels of stress hormones postoperatively. On the other hand, based on mRNA expression changes recorded in RA during major abdominal surgery [4] it could be suggested that trauma mediated induction of inflammatory pathways in skeletal muscle *per se*, rather than circulating cytokines, may be an important driver of postoperative insulin resistance. Indeed, Witasp et al. [4] demonstrated increased expression of mRNAs encoding inflammation proteins such as IL-6 and TNF-α in skeletal muscle during major abdominal surgery. Importantly, as far as we are aware no study has documented muscle inflammatory mRNA and protein and tissue metabolic responses both locally and distant from the site of surgical trauma. Here, we have shown that major abdominal surgery was associated with elevated plasma IL-6, TNF-α and cortisol concentrations, which persisted for 48 h in the case of IL-6 (Table 2). In tandem with this, mRNA expression of IL-6 and TNF-α was profoundly elevated in RA (>1000 fold and >15 fold, respectively) and VL (∼100 fold and ∼6 fold, respectively) immediately after surgery compared with at the start of surgery (Fig. 2). Based on these data, and in keeping with the sentiment of Witasp et al. [4], one could reasonably postulate postoperative impairment of blood glucose disposal is, at least partly, attributable to the cytokine mediated impairment of muscle glucose uptake. In line with this, Fig. 3 illustrates that the increase in muscle mRNA abundance of IL-6 and PDK4 we observed was parallelled by increased expression at the protein level in the RA immediately after surgery. Increasing evidence suggests that activation (dephosphorylation) of the FOXO family of transcription factors plays an important part in triggering the inhibition of muscle carbohydrate oxidation in metabolic stress situations, at least in part, through transcriptional upregulation of its downstream target PDK4, and consequently decreased PDC activation and flux (the rate-limiting step in the carbohydrate oxidation), thereby resulting decreased oxidative glucose utilisation and elevated tissue glycogen use and lactate content [26], [27]. Furthermore, we have previously provided substantive evidence of muscle cytokine accumulation mediating these events during LPS-induced endotoxaemia in rodents [10], [12], [28], and in critically-ill patients [29]. Importantly, the postoperative elevation in muscle IL-6 and PDK4 protein content in RA was accompanied by reduced muscle glycogen and muscle lactate contents (Table 3), strongly suggesting the induction of muscle metabolic dysregulation at this time point. Given that a similar, albeit less marked, muscle response was observed in the VL postoperatively, it is plausible that this may have resulted in similar metabolic and protein changes over the course of 48 h postoperative recovery to those observed in the RA immediately after surgery. We, therefore, speculate that muscle-specific inflammation and metabolic dysregulation, particularly local to the site of surgery, may be contributing to the well documented impaired glucoregulation in the days following surgery. Importantly, although these changes were accompanied by an increase in postoperative gut permeability, we found no evidence of bacteraemia 48 h postoperatively, making it unlikely that bacterial translocation across the gut contributed to the systemic or muscle level responses we have reported.

Cytokines also influence skeletal muscle protein catabolism by directly modulating protein synthesis and degradation and indirectly, through inhibition of the action of regulatory anabolic hormones [30] and activation of hypothalamic–pituitary–adrenal axis [31]. In keeping with this, we have previously shown that muscle inflammation induced atrophy during LPS-induced endotoxaemia in rodent skeletal was tightly coupled to the upregulation of the FOXO downstream targets MAFbx, MuRF1, 20S proteasome and cathepsin-L at both mRNA and protein levels [10], [12], and a similar pattern of events also evident in the muscle of intensive care patients [29]. The muscle specific E3-ubiquitin ligases MAFbx and MuRF1 [32], are considered to be key regulators of ubiquitin proteasome mediated muscle protein degradation [6], and are dampened using anti-inflammatory interventions [10], [11]. In keeping with this, we were also able to show substantial upregulation of MAFbx mRNA (Fig. 2) in RA and VL immediately after surgery, which was accompanied by increase protein expression in the RA (Fig. 3). Again, despite evidence of increased gut permeability 48 h postoperatively (Fig. 1A), these muscle level changes immediately after surgery were not supported by the presence of bacteraemia 48 h later (Fig. 1B), suggesting once more that muscle level inflammation secondary to surgical stress was driving cellular events and upregulating muscle protein breakdown.

Surgical stress was associated with increased mRNA expression of FOXO1 in RA and VL and several FOXO gene targets (Fig. 2). However, the finding that FOXO1 protein phosphorylation was not decreased relative to at the start of surgery, particularly in the RA, was unexpected (Fig. 3). There is no obvious explanation for this observation given the increased expression of both PDK4 and MAFbx protein in the RA, one explanation is the increase in RA cytokine protein expression mediated effects on FOXO downstream targets via FOXO3 phosphorylation, rather than FOXO1. LPS induced increases in muscle cytokines has been shown to be parallelled by decreased cytosolic FOXO1 and FOXO3 phosphorylation in rodent endotoxaemia [12].

To conclude, surgical trauma induced a systemic proinflammatory cytokine response immediately after surgery that was accompanied by an increase in gut permeability, but there was no evidence of bacteraemia 48 h later. This systemic inflammatory response to surgical trauma, in the absence of bacteraemia or sepsis, was accompanied by profound muscle cytokine mRNA responses and metabolic dysregulation immediately post-surgery, particularly local to the site of surgical trauma, which was consistent with the impairment of post-prandial glucoregulation recorded 48 h postoperatively. Collectively, these findings suggest that muscle level responses, but not the presence of bacteraemia, likely contribute to the impairment of muscle mass maintenance and systemic blood glucose regulation in the days following major abdominal surgery.

## Conflicts of interest

None of the authors has a direct conflict of interest to report.

DN Lobo has received unrestricted research funding, educational grants and speakers' honoraria from BBraun and Baxter Healthcare Inc. for unrelated work.

## Author contributions

KKV – study design, literature search, data collection, data analysis, data interpretation, writing of the manuscript and final approval.

DT-C – study design, literature search, data collection, conducting experiments, data analysis, data interpretation, figures, writing of the manuscript and final approval.

DC – study design, conducting experiments, data analysis, data interpretation, writing of the manuscript and final approval.

PLG – study design, literature search, data interpretation, writing of the manuscript, critical review, supervision and final approval.

DNL – study design, literature search, data interpretation, figures, writing of the manuscript, critical review, supervision and final approval.
